# Role of PKCtheta in macrophage-mediated immune response to *Salmonella typhimurium* infection in mice

**DOI:** 10.1186/s12964-016-0137-y

**Published:** 2016-07-28

**Authors:** Christa Pfeifhofer-Obermair, Karin Albrecht-Schgoer, Sebastian Peer, Manfred Nairz, Kerstin Siegmund, Victoria Klepsch, David Haschka, Nikolaus Thuille, Natascha Hermann-Kleiter, Thomas Gruber, Günter Weiss, Gottfried Baier

**Affiliations:** 1 Department for Pharmacology and Genetics, Division of Translational Cell Genetics, Peter Mayr Straße 1a, 6020 Innsbruck, Austria; 2 Department of Internal Medicine VI/Infectious Diseases, Immunology, Rheumatology, Pneumology, Medical University Innsbruck, Anichstraße 35, 6020 Innsbruck, Austria

**Keywords:** *Salmonella typhimurium*, Protein kinase C theta, Innate immunity, Macrophage polarization, IL-10

## Abstract

**Background:**

The serine/threonine protein kinase C (PKC) theta has been firmly implicated in T cell-mediated immunity. Because its role in macrophages has remained undefined, we employed *PKCtheta*-deficient (*PKCtheta*^−/−^) mice in order to investigate if PKCtheta plays a role in macrophage-mediated immune responses during bacterial infections.

**Results:**

Our results demonstrate that PKCtheta plays an important role in host defense against the Gram-negative, intracellular bacterium *Salmonella typhimurium*, as reflected both by markedly decreased survival and a significantly enhanced number of bacteria in spleen and liver of *PKCtheta*^−/−^ mice, when compared to wild-type mice. Of note, albeit macrophages do not express detectable PKCtheta, *PKCtheta* mRNA expression was found to be profoundly upregulated during the first hours of lipopolysaccharide (LPS)/interferon-gamma (IFNgamma)-, but not IL-4-mediated cell polarization conditions in vitro. Mechanistically, despite expressing normal levels of classically activated macrophage (CAM) markers, *PKCtheta*-deficient CAMs expressed significantly higher levels of the anti-inflammatory cytokine IL-10 in vivo and in vitro when challenged with *S. typhimurium* or LPS/IFNgamma. Neutralization of IL-10 recovered immune control to *S. typhimurium* infection in *PKCtheta*-deficient macrophages.

**Conclusions:**

Taken together, our data provide genetic evidence that PKCtheta promotes a potent pro-inflammatory CAM phenotype that is instrumental to mounting protective anti-bacterial immunity. Mechanistically, PKCtheta exerts a host-protective role against *S. typhimurium* infection, and acts as an essential link between TLR4/IFNgammaR signaling and selective suppression of the anti-inflammatory cytokine IL-10 at the onset of CAM differentiation in the course of a bacterial infection.

## Background

Macrophages (Mφ) play important roles in inflammatory and infectious diseases. Mφ polarization phenotypes, however, are heterogeneous and profoundly affected by local factors within the microenvironment. Microbial stimuli such as lipopolysaccharide (LPS), interleukin-1beta (IL-1beta), and cytokines secreted by Th1 lymphocytes, such as interferon-gamma (IFNgamma) induce a “classic” Mφ phenotype (CAM). Activated CAMs are pro-inflammatory Mφ and produce high levels of pro-inflammatory cytokines like tumor necrosis factor-alpha (TNF-alpha), IL-6, IL-1beta, IL-23 and IL-12. CAMs are essential components of anti-microbial host defense and further characterized by production of reactive oxygen species and reactive nitrogen species during the promotion of a Th1-type driven response [1–3]. Because these pro-inflammatory CAMs may cause extensive tissue damage, their activation is tightly controlled. Indeed, CAMs are established to be involved in autoimmune diseases such as systemic lupus erythematosus and rheumatoid arthritis [4]. In contrast, Th2 lymphocyte cytokines such as IL-4 and IL-13 promote the alternatively activated Mφ phenotype (AAM) that dampens the inflammatory state by producing anti-inflammatory mediators such as IL-10. Signature genes for AAMs are Arginase-1, chitinase-like molecules YM1 and YM2, and resistin-like molecule Fizz-1 (Relm-alpha, Retnla) [5, 6]. AAMs, in particular, produce high amounts of the anti-inflammatory cytokines IL-10 and Tumor growth factor beta (TGFβ), thereby inhibiting pro-inflammatory immune responses. Thus, AAMs are involved in tissue remodelling [7] and tumor progression [8].

Protein kinase C (PKC) isotypes are members of the serine/threonine protein kinase subfamily, and play an important role in the regulation of a variety of cell functions. PKCtheta (PKCtheta), a member of the nPKC subfamily, is predominantly expressed in T cells. Most of our current knowledge about PKCtheta therefore arises from studies performed in T cells. We and others could show that PKCtheta plays a critical role in the NF-kappaB, AP-1, and Ca^2+^/NFAT pathways to activate e.g. the interleukin-2 cytokine promoter [9, 10]. Madaro et al. demonstrated that PKCtheta has an immune cell intrinsic role in muscle tissue repair during muscular dystrophy inflammation [11]. More recently, Ma et al. reported a role for PKCtheta in cholesterol metabolism in human Mφ [12]. Because different PKCs, namely PKCalpha, PKCbeta, PKCdelta and PKCzeta, have been shown to have a critical role in Mφ antimicrobial immune responses [13–16], we investigated the functional role of PKCtheta in Mφ biology. Here we demonstrate that the genetic ablation of PKCtheta expression in mice leads to exacerbation of disease progression and early death in *Salmonella (S.) typhimurium* infection.

## Results

### *PKCtheta* mRNA expression is selectively induced in LPS/IFNgamma polarizing conditions

**Fig. 1:**
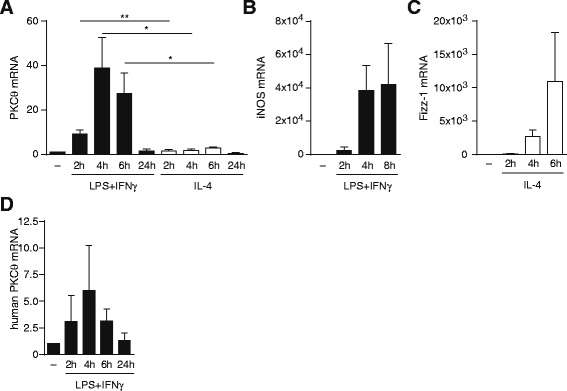
*PKCtheta* mRNA levels are transiently induced in an LPS/IFNgamma-dependent manner in both mouse and human macrophages: **a** BMDMs were differentiated and polarized to CAMs (LPS/IFNgamma) and AAMs (IL-4). *PKCtheta* gene expression was determined by qRT-PCR after different time points and was found to be selectively expressed under CAM polarization. Data shown are derived from at least three independent experiments and qRT-PCR data were normalized to Gapdh. Error bars denote the mean ± s.e.m. *p < 0.05; ** *p* < 0.01; Expression of unstimulated macrophages was arbitrarily set to 1. **b** + **c** In order to control proper polarization of macrophages, gene expression of prominent markers (iNOS for CAM, Fizz-1 for AAM) were validated. **d** CD14+ human monocytes from peripheral blood were differentiated and polarized with LPS/IFNgamma. After predetermined time periods, gene expression of *PKCtheta* was analysed with qRT-PCR, revealing an increase in *PKCtheta* mRNA levels upon stimulation in the human setting, consistent with results from mouse experiments. Data shown are relative to expression of unstimulated macrophages, which was arbitrarily set to 1. All data were normalized to *Gapdh*

PKCtheta has been reported to regulate the expression of various genes in T cells [17]. To examine whether PKCtheta modulates Mφ polarization in pro- or anti-inflammatory milieu conditions, we analysed mRNA expression patterns of PKCtheta in bone marrow-derived macrophages (BMDM) polarized with lipopolysaccharide (LPS)/interferon-gamma (IFNgamma) or with IL-4, respectively (Fig. 1a). For proper polarization, marker gene expression of iNOS (for CAM) and Fizz-1 (for AAM) was monitored (Fig. 1b + c). As a remarkable result, PKCtheta expression was strongly induced in LPS/IFNgamma-polarized but not in IL-4-polarized cells. Of note, *PKCtheta* mRNA up-regulation in CAMs peaked at 4 h. These results suggest a subset-selective role for PKCtheta as “signaling intermediate” of pro-inflammatory Mφ. Reminiscent to mouse cells, PKCtheta is inducibly expressed in human CD14+ monocytes polarized with LPS/IFNgamma, indicating a possible role of PKCtheta also in fine-tuning of the cellular phenotype of human cells (Fig. 1d).

### PKCtheta is causally involved in the protection from peritoneal *S. typhimurium* infection

**Fig. 2:**
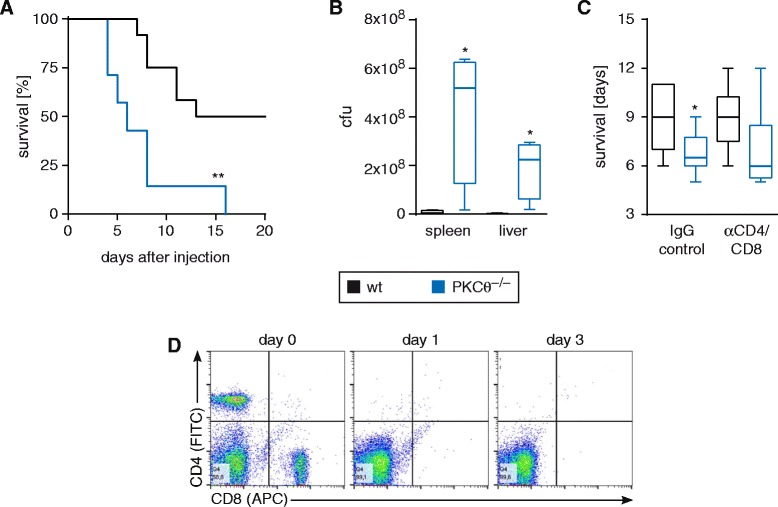
*PKCtheta* ^−/−^ mice show markedly decreased survival and increased number of colony-forming units in spleen and liver compared to wild-type mice. **a** Survival studies of wild-type (WT) versus *PKCtheta* ^−/−^ mice revealed a significant disadvantage of *PKCtheta*-deficient animals after intraperitoneal injection of 50,000 cfu of *S. typhimurium* depicted by a Kaplan-Meier curve and statistically analysed by the log-rank test (*p* = 0.0024). **b** Bacterial loads in spleen and liver were determined on day 4 after infection with *S. typhimurium* and displayed a significant increase in cfu in *PKCtheta* ^−/−^ mice. Data are expressed as mean ± s.e.m. *p < 0.05; **c** In order to investigate the role of T cells in the survival disadvantage of *PKCtheta* ^−/−^ mice after infection with *S. typhimurium*, CD4^+^ and CD8^+^ T cells were depleted with the corresponding antibodies prior to infection. IgG isotype antibodies were used for control animals. Results show no difference in survival in T cell-depleted mice versus mice with IgG control, indicating a negligible role of CD4^+^ and CD8^+^ T cells in the reduced survival rate of *PKCtheta* ^−/−^ mice. Data are expressed as mean ± s.e.m. *p < 0.05; **d** Depletion of CD4^+^ and CD8^+^ T cells was controlled by FACS analysis on day 0 (prior to depletion), day 1 (infection with *S. typhimurium*) and day 3. Antibodies and IgG controls were injected every third day during the course of the experiment

Based on significant PKCtheta expression level in CAMs, we investigated its potential role in bacterial killing, employing infection assays with intracellular Gram-negative *S. typhimurium* bacteria in vivo and *ex vivo*. Evaluation of wild-type and *PKCtheta*^−/−^ mice during systemic infection showed that all of the *PKCtheta*^−/−^ mice died within 16 days whereas a minimum of 50 % wild-type mice survived, suggesting a critical role for PKCtheta in the host defense against *S. typhimurium* (Fig. 2a). As *PKCtheta*^−/−^ mice started to die around day 4–5, we monitored the bacterial load in spleen and liver at day 4 which revealed a significant increase in the number of colony-forming units (cfu) of *S. typhimurium* in *PKCtheta*^−/−^ mice (Fig. 2b). These data clearly establish an essential and non-redundant positive role of PKCtheta in protection from *S. typhimurium*.

### T cells are not primarily responsible for the reduced survival of *PKCtheta*^−/−^ mice after peritoneal infection with *S. typhimurium*

To investigate whether T cells contribute to the significantly reduced survival phenotype of *PKCtheta*^−/−^ mice during *S. typhimurium* infection, we depleted CD4^+^ and CD8^+^ T cells with antibodies before injection of *S. typhimurium* (Fig. 2c). Depletion antibodies were re-injected every 3 days during the course of the experiment. Isotype-matched antibodies were injected as depletion control and effective depletion was controlled by FACS analysis (Fig. 2d). The results showed no significant difference in survival of mice injected either with CD4 and CD8 depletion antibodies or isotype control, indicating that T cells are not primarily responsible for the reduced survival rate of *PKCtheta*^−/−^ mice. These data suggest that *PKCtheta*^−/−^ mice fail to mount the appropriate innate immune responses after infection with *S. typhimurium*.

### PKCtheta plays a crucial role in repression of IL-10 production in vitro and in vivo

**Fig. 3:**
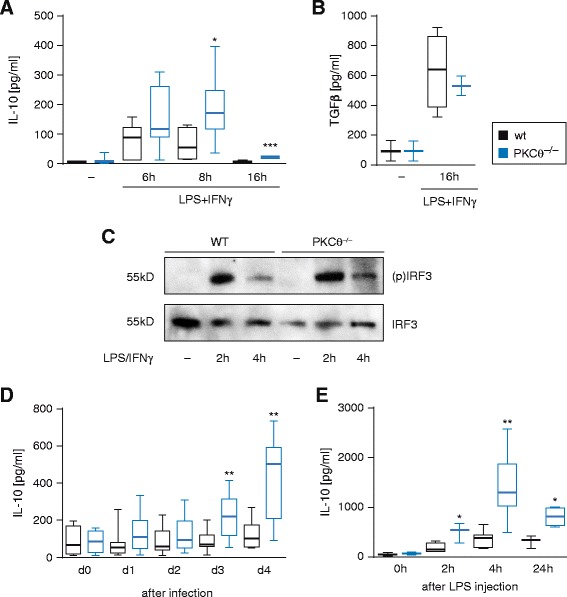
*PKCtheta* deficiency leads to IL-10 hyperproduction of bone marrow-derived macrophages. **a**, **b** Bioplex technology was used to analyse secretion responses of the anti-inflammatory cytokines IL-10 (**a**) and TGFbeta (**b**) in the supernatant of differentiated and LPS/IFNgamma-polarized macrophages from *PKCtheta* ^−/−^ versus WT mice. Error bars show the mean of at least three independent experiments ± s.e.m. *p < 0.05; ***p < 0.001; **c** Differentiated BMDM were allowed to rest for 1 h in X-Vivo 20 medium and were then stimulated with LPS/IFNgamma (100 ng/ml and 10 ng/ml, respectively) for 2 and 4 h and whole cell lysates were subjected to immunoblotting against phosphorylated IRF3 and total IRF3. **d** Analysis of IL-10 serum concentration of WT and *PKCtheta* ^−/−^ mice after infection with *S. typhimurium* from day 0 to day 4 with Bioplex technology. Error bars show the mean of at least three independent experiments ± s.e.m. **p < 0.01; **e** IL-10 serum levels from peripheral blood of wild-type featuring *PKCtheta* ^−/−^ mice after challenging mice with intraperitoneal LPS injection as an alternative infection model. Data were collected from at least three experiments and shown as mean ± s.e.m. *p < 0.05; **p < 0.01

In T cells, PKCtheta is known to affect a wide range of transcription factors such as AP-1, NF-kappaB and NFAT, regulating i.e. IL-2 activation responses. Additionally, PKCtheta influences the transcription of miRNA clusters [18] and, in turn, controls expression of many other genes. To investigate a PKCtheta signaling function upstream of cytokine responses in Mφ, LPS/IFNgamma-polarized BMDMs from wild-type and *PKCtheta*^*−/−*^ mice were monitored for pro- and anti-inflammatory cytokine production at different time points. Although we could not detect any differences in the production of IL-6, IL-12p40, TNF-alpha as well as iNOS expression (data not shown) in polarized *PKCtheta*^−/−^ CAMs, we found a significantly increased production of the anti-inflammatory cytokine IL-10 in vitro (Fig. 3a). Of, note, the anti-inflammatory cytokine TGFβ response of polarized *PKCtheta*^−/−^ CAMs, remained unchanged both at the protein and the mRNA levels (Fig. 3b and data not shown), indicating a selectivity of PKCtheta signaling function. Mechanistically and albeit our results do not rule out effects of *PKCtheta* deficiency on TLR4 signaling responses in general, PKCtheta does not regulate TLR4 signaling leading to IRF3 activation response (Fig. 3c). In agreement with this observation, the significantly lowered survival rates of *PKCtheta*^−/−^ mice during peritoneal *S. typhimurium* infection correlated with significantly higher IL-10 serum levels (Fig. 3d). Additionally, after injecting LPS intraperitoneally, the anti-inflammatory IL-10 serum response in *PKCtheta*-deficient mice was profoundly increased after 2 to 24 h (Fig. 3e). This further confirms the increased IL-10 production by myeloid cells in another in vivo model. Thus, although Mφ from either *PKCtheta*^−/−^ or wild-type mice were polarized into CAMs, only wild-type Mφ but not *PKCtheta*-deficient Mφ mounted a functional anti-bacterial activity.

### PKCtheta-mediated IL-10 repression affects bacterial killing of macrophages in vitro

**Fig. 4:**
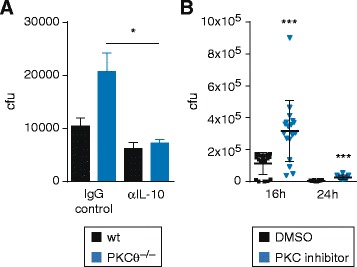
PKCθ is involved in bacterial killing of macrophages after infection with *S. typhimurium* via inhibition of IL-10 deregulation. **a** Killing capacity of BMDMs from WT versus *PKCtheta* ^−/−^ mice was investigated after infection of macrophages with *S. typhimurium*. Macrophages were treated with IL-10 or IgG control antibodies. Significantly enhanced numbers of colony-forming units (cfu) were found in lysates of macrophages from *PKCtheta* ^−/−^ mice compared to WT mice. This effect could be abrogated with IL-10 blockade. Data represent the mean of at least three independent experiments ± s.e.m. *p < 0.05; **b** PKC inhibitor AEB071 enhanced cfu in macrophages infected with *S. typhimurium* highly significantly as shown in a killing experiment with BMDM from WT mice. DMSO served as control vehicle. Error bars denote the mean ± s.e.m. ***p < 0.001

Because containment and killing of *S. typhimurium* after invasion of Mφ is a key event in host protection, we monitored intracellular bacterial loads of *S. typhimurium*-infected Mφ of both genotypes, reproducibly demonstrating increased bacterial numbers in *PKCtheta*^−/−^ CAMs as compared to wild-type macrophages (Fig. 4a). In order to investigate a direct role of IL-10 hyper-production for the reduced killing capacity of Salmonella-infected *PKCtheta*^−/−^ Mφ, IL-10 was neutralized with IL-10 blocking- or isotype control antibodies. We observed that IL-10 blocking antibody treatment rescued the killing capacity of *PKCtheta*-deficient cells back to the wild-type level (Fig. 4a). Similar to genetic *PKCtheta* depletion, pharmacological PKCtheta inhibition by the PKC-selective inhibitor AEB071 (Sotrastaurin) [19] significantly reduced the killing capacity of *S. typhimurium*-infected wild-type Mφ (Fig. 4b). Taken together, these results suggest that PKCtheta is critically involved in bacterial killing of *S. typhimurium*-infected Mφ by suppressing IL-10 production.

## Discussion

*S. typhimurium* is a Gram-negative, motile, facultative intracellular bacterium, which invades and multiplies within mononuclear phagocytic cells in liver, spleen, lymph nodes, and Peyer’s plaques [20, 21]. *S. typhimurium* causes severe gastrointestinal disorders in humans and typhoid fever with systemic infections in mice. Mφ functions have been strongly implicated in infectious diseases. Mφ, as one of the first barriers of the innate immune system, rapidly control *S. typhimurium*. Importantly, however, these bacteria may evade immune control and even multiply within Mφ by mechanisms that are insufficiently understood so far [22, 23].

The immune response to bacterial infection is regulated by the counter-play between different Mφ subsets, namely CAMs and AAMs. Changes in the balance of CAM- and AAM- subsets and their effector cytokines result in alterations of disease progression. Recognition of Gram-negative bacteria in Mφ involves the binding of LPS to TLR4, leading to secretion of key cytokines [24, 25]. Furthermore, Mφ produce ROS by the phagocyte NADPH oxidase (NOX2; phox) and RNS by the inducible nitric oxide synthase (iNOS; NOS2) to combat bacteria. To counteract chronic pro-inflammatory immune effector mechanisms, the production of anti-inflammatory proteins IL-10 and TGFbeta by Mφ is induced [26, 27]. NO, generated by IFNgamma-induced iNOS, is shut down with a shift to Arg1, and YM1 and YM2 are strongly induced, subsequently leading to the induction of a Th2 immune response [28].

IL-10 plays an essential part in dampening inflammation [29]. Consequently, dysregulation of IL-10 is linked with susceptibility and an impaired clinical course of numerous infections in mouse models and in humans [30–32]. Mechanistically, distinct signaling and epigenetic chromatin remodelling of the *IL10* locus control the production of IL-10. Furthermore, post-transcriptional events through e.g. microRNA represent a critical step for IL-10 transcript stability.

In response to LPS, TLR4 mediated signaling utilizes IRF3 to induce an IL-10 transcription response. Nevertheless, a number of additional transcription factors including signal transducers and activators of transcription (STAT), activator protein (AP), cAMP response element binding protein (CREB), CCATT enhancer/binding protein (C/EBP), c-musculoaponeurotic fibrosarcoma factor (c-MAF), and nuclear factor kappa B (NF-kappaB), have been characterized as essential or critical in myeloid cell-type-specific IL-10 gene regulation. Thus a comprehensive understanding of the detailed cellular processes of PKCtheta that contribute in a CAM cell-specific manner to IL-10 transcriptional regulation remains elusive. Albeit we cannot define the exact signaling function of PKCtheta upstream of *Il10* transcriptional regulation, the results are in a line with our observation of increased IL-10 formation in *PKCtheta*-deficient BMDMs and that neutralization of IL-10 results in an improved control of bacterial proliferation that is indistinguishable between wild-type and *PKCtheta*-deficient macrophages.

## Conclusions

We demonstrate that PKCtheta contributes to a host-protective immune response against peritoneal *S. typhimurium* infection by its selective involvement during early (up to 24 h) pro-inflammatory CAM polarization. This time frame is in agreement with the fact that in these cells PKCtheta is induced immediately and transiently after LPS/IFNgamma engagement. *PKCtheta* deficiency, as defined in this study, leads to an altered CAM phenotype, based on an incomplete suppression of the macrophage deactivating cytokine IL-10. The detailed molecular basis of PKCtheta-dependent regulation of IL-10 production under CAM-promoting conditions, and thus the phenotypic instability of *PKCtheta*^−/−^ CAMs, however, remains unknown. Nevertheless, the data define a previously unknown regulatory role of PKCtheta in macrophage driven innate defense mechanisms against bacterial infections. As an innovative paradigm, CAM-intrinsic PKCtheta may represent a transiently inducible signaling intermediate leading to IL-10 suppression, and subsequently, to an effective anti-bacterial immunity against intracellular bacteria such as *S. typhimurium*. This hypothesis is based on the increased IL-10 expression observed during the *PKCtheta*^*−/−*^ CAM-priming phase. Of note, this deregulated IL-10 expression level observed in *PKCtheta*^−/−^ CAMs is directly responsible for their bacterial killing defects after infection with *S. typhimurium*.

In summary, the findings of this study provide genetic evidence that PKCtheta is an early factor involved in developing a stable CAM-mediated immune response in vitro and in vivo*.* Our results provide first experimental evidence for an involvement of PKCtheta in Mφ biology, a finding relevant for the understanding of PKCtheta in innate anti-microbial immune effector function.

## Methods

### Mice

Wild-type and *PKCtheta*^−/−^ mice (on mixed C57BL/6x129/Sv background) were maintained under specific pathogen-free conditions in the central animal facility of the Medical University of Innsbruck. *PKCtheta*^−/−^ mice have previously been described in detail (3). All animal experiments have been performed in accordance with national and European guidelines and have been reviewed and authorized by the committee on animal experiments (Federal Ministry of Science, Research and Economy-66.0ll/0128-WF/V/3b/2014).

### Cell culture

Bone marrow-derived macrophages (BMDM) were harvested from tibiae and femora of 8 to 12-week-old mice and differentiated for 7 days in complete DMEM (Biochrom) supplemented with 10 % FCS, 2 mM L-glutamine, 10,000 U/mL penicillin plus 10 mg/mL streptomycin (all from Biochrom) and 15 % L929 supernatant. Medium was replaced after 4 days. On day 7, cells were washed with phosphate-buffered saline (PBS) and polarized with either 10 ng/ml LPS (L6511; Sigma-Aldrich) and 10 ng/ml IFNgamma (BMS326, eBioscience) or with 10 ng/ml IL-4 (14-8041-62; eBioscience) for different time periods in X-Vivo 20 medium (BE04-448Q, Lonza).

### Western blotting

BMDM (1 × 10^6^) were lysed in lysis buffer (50 mM Tris–HCl, pH 7.3, 5 mM NaF, 5 mM Na_3_VO_4_, 5 mM NaP_2_P, 5 mM EDTA, 50 mM NaCl, 1 % NP-40, 50 μg/ml aprotinin, 50 μg/ml leupeptin) and subjected to SDS-PAGE on Bis/Tris-buffered gels (Novex). After transfer to nitrocellulose membrane by semi-dry blotting, Ser-396 phosphorylated as well as pan-IRF3 were detected by immune blotting (both with antibodies from Cell Signaling Technology).

### Gene expression analysis

Total RNA was isolated using the RNeasy® Mini Kit (Qiagen) according to the manufacturer’s instructions. cDNA was synthesized using the Qiagen Omniscript RT Kit and oligo(dT) primers (Promega). Expression analysis was performed with real-time PCR using TaqMan technology (assays from Applied Biosystem). Reactions were run with Applied Biosystems 7500 Fast Sequence Detection System. All gene expressions were normalized to Gapdh.

### Human CD14+ monocyte polarization

Ficoll-Paque Premium (17-5442-02; GE-Life Sciences) was used to isolate mononuclear cells from blood of healthy volunteers according to the manufacturer’s instructions. CD14+ cells were positively selected with human CD14 MicroBeads (130-050-201; MACS Miltenyi Biotec) and cultured in DMEM (BioWhittaker BE12-707 F, Lonza) supplemented with 10 % FCS, 2 mM L-glutamine, 1 % penicillin plus streptomycin (10,000 U/mL penicillin and 10 mg/mL streptomycin in 0.9 % NaCl), and 100 ng/ml of human GM-CSF (572903, BioLegend). Cells were fed on day 4 and stimulated with 10 ng/ml LPS (lipopolysaccharide from *S. typhimurium* L6511; Sigma-Aldrich, Vienna, Austria) and 10 ng/ml human IFNgamma (570204, BioLegend) on day 7 for the indicated time periods.

### Bacteria-induced infection model

8 to 12-week-old male WT and *PKCtheta*^−/−^ mice were infected intraperitoneally with 50,000 cfu of *Salmonella enterica serovar typhimurium* (ATCC14028) diluted in 200 μl PBS, and survival was monitored over 21 days. For cytokine measurement, blood samples were taken on day 0 to day 4 and serum was collected. Bacterial load of organs was determined by plating serial dilutions of organ homogenates from day 4 after infection on LB broth agar (L7275-500TAB; Sigma-Aldrich) under sterile conditions and the number of bacteria was calculated per gram of tissue after cultivation.

### In vivo T cell depletion

Mice were injected with 500 μg of anti-mouse CD4 (clone GK1.5; BE003-1) and anti-mouse CD8 antibody (clone YTS 169.4; BE0117), or the corresponding IgG2b (clone LTF-2; BE0090) control (all from BioXCell, USA) 1 day prior to infection with *S. typhimurium*. Facial vein blood was taken after 24 h to control T cell depletion by FACS analysis using antiCD4-FITC, antiCD8-APC, and antiCD3-PE (all from eBioscience). Thereafter, 5,000 to 30,000 cfu *S. typhimurium* were injected intraperitoneally. 300 μg of depletion antibodies or isotype control were administered every third day throughout the entire experiment.

### Cytokine measurements

IL-10 and TGFbeta cytokine levels from from serum or cell culture supernatants were analyzed with Bio-Plex multianalyte technology (BioRad).

### Endotoxin-induced infection model

WT and *PKCtheta*^−/−^ mice were treated with intraperitoneal injection of 15 mg LPS (lipopolysaccharide from *S. typhimurium* L6511; Sigma-Aldrich) per kg of body weight. Serum was collected 4 h after LPS challenge for analysis of IL-10.

### Salmonella infection in vitro

Macrophages were isolated as described above and incubated in complete DMEM without antibiotics. Wild-type strain *S. typhimurium* was cultured in LB broth to late-logarithmic phase. Mφ were infected with *S. typhimurium* at a multiplicity of infection (MOI) of 10 for 1 h. Thereafter, cells were washed with PBS and incubated in complete DMEM containing gentamycin (Gibco). For killing experiments, prior to infection, cells were treated with a monoclonal rat anti-mouse IL-10 antibody (10 μg/ml; clone JES5-2A5; 504903; BioLegend) or the appropriate isotype control (10 μg/ml; clone RTK2071; 400413; BioLegend) for 23 h. AEB071 (sotrastaurin) was used as PKC inhibitor in killing experiments and cells were treated with 1 μM for 23 h. After infection, intracellular bacterial loads were harvested with 0.5 % sodium deoxycholic acid (D6750; Sigma-Aldrich) as described previously [33].

## Abbreviations

AAM, alternatively activated macrophages; CAM, classically activated macrophages; LPS, lipopolysaccharide; Mφ, macrophages; PKC, Protein kinase C; S., salmonella
